# Catamenial Pneumothorax in a Patient Undergoing Low-Dose Estrogen-Progestin Therapy: A Case Report

**DOI:** 10.7759/cureus.75527

**Published:** 2024-12-11

**Authors:** Naoya Ishibashi, Hiromichi Niikawa, Ryuga Yabe, Ryo Nonomura, Yutaka Oshima, Takanobu Sasaki, Takafumi Sugawara

**Affiliations:** 1 Thoracic Surgery, Tohoku Medical and Pharmaceutical University, Sendai, JPN

**Keywords:** catamenial pneumothorax, low-dose estrogen-progestin therapy, pneumothorax (ptx), secondary pneumothorax, video-assisted thoracic surgery

## Abstract

A 46-year-old woman on low-dose estrogen-progestin (LEP) therapy for endometriosis developed a right-sided pneumothorax. Surgical findings included a pulmonary bulla in the right middle lung lobe and a small hole in the center tendon of the diaphragm, both of which were partially resected. Histopathology confirmed the presence of endometrial tissue, leading to a diagnosis of thoracic endometriosis. This case demonstrates that catamenial pneumothorax can occur despite LEP therapy, which is intended to suppress endogenous hormones. Clinicians should remain vigilant for this condition in patients with a history of endometriosis, even when hormonal treatment is in use.

## Introduction

Catamenial pneumothorax (CP) is a subtype of secondary spontaneous pneumothorax that predominantly affects women of reproductive age and is closely associated with thoracic endometriosis [1]. The condition warrants careful attention due to its complex pathogenesis, high recurrence rates, and challenges in diagnosis. The proposed mechanisms regarding when and how endometrial tissue might reach the thoracic cavity include embryonic migration and postnatal mechanisms, such as transperitoneal spread and hematogenous seeding [2,3]. Treatment of CP typically involves a combination of surgery and hormonal therapy. Hormonal therapy is essential to control endogenous hormone levels and reduce recurrence rates. Surgical intervention, including partial resection of affected lung and diaphragmatic tissue, serves both diagnostic and therapeutic purposes, especially in cases where the diagnosis cannot be confirmed by preoperative imaging. Despite the efficacy of low-dose estrogen-progestin (LEP) therapy in stabilizing the menstrual cycle and reducing symptoms of endometriosis, this case demonstrates that CP can occur even under hormonal suppression, requiring a multidisciplinary approach to treatment and long-term management. In this report, we present a case of CP in a patient who had received LEP therapy and emphasize the importance of early recognition, appropriate surgical intervention, and the role of postoperative hormonal management in preventing recurrence.

## Case presentation

**Figure 1:**
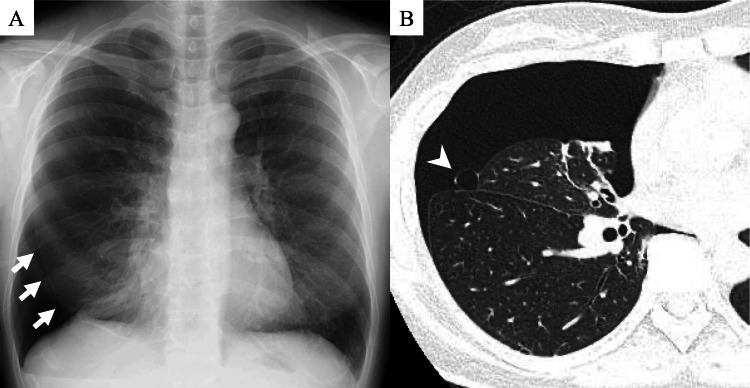
Chest X-ray (A) and chest computed tomography (B). A: Chest X-ray showing a right pneumothorax (arrow). B: Chest computed tomography showing bulla in the right middle lung lobe (arrowhead).

A 46-year-old woman with a 22-year history of endometriosis, initially diagnosed through laparoscopic findings of endometrial implants in the pelvic peritoneum, including the ovarian surface and Douglas pouch, underwent diagnostic laparoscopy followed by cyclic LEP therapy (21 days on, seven days off), consisting of norethisterone 1 mg and ethinyl estradiol 0.02 mg. She had no prior history of pneumothorax and a 2.5 pack-year smoking history. The last menstrual period occurred eight days before the onset of symptoms. Three days after discontinuing LEP, she developed sudden right back pain at home and was kept under observation. Four days later, she resumed LEP; however, her pain worsened, and she experienced exertional dyspnea. She subsequently visited her local physician, who diagnosed a right pneumothorax via chest X-ray and referred her to our department (Figure 1A).

Chest computed tomography (CT) revealed a pulmonary cyst in the right middle lobe and moderate right lung collapse (Figure 1B).

**Figure 2:**
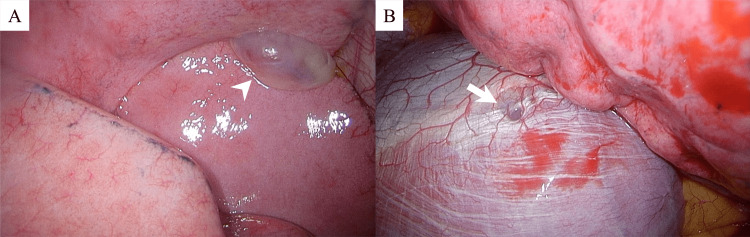
Intraoperative findings (A: lung; B: diaphragm). A: Intraoperative findings showing bulla in the middle lung lobe (arrowhead). B: A small hole in the central tendon of the diaphragm (arrow).

A thoracic drain was inserted. Due to a persistent pulmonary fistula, she was referred for surgery. The operation was performed thoracoscopically with three ports. We identified a pedunculated cyst in the middle lobe of the right lung and a small hole in the center of the diaphragmatic tendon (Figure 2). An air leakage test confirmed leakage from the bulla, but no redness or abnormalities were observed on its wall or base.

The procedure included partial resection of the lung cyst and diaphragm using automatic suturing. The chest tube was removed on postoperative day one, and the patient was discharged on postoperative day three.

**Figure 3:**
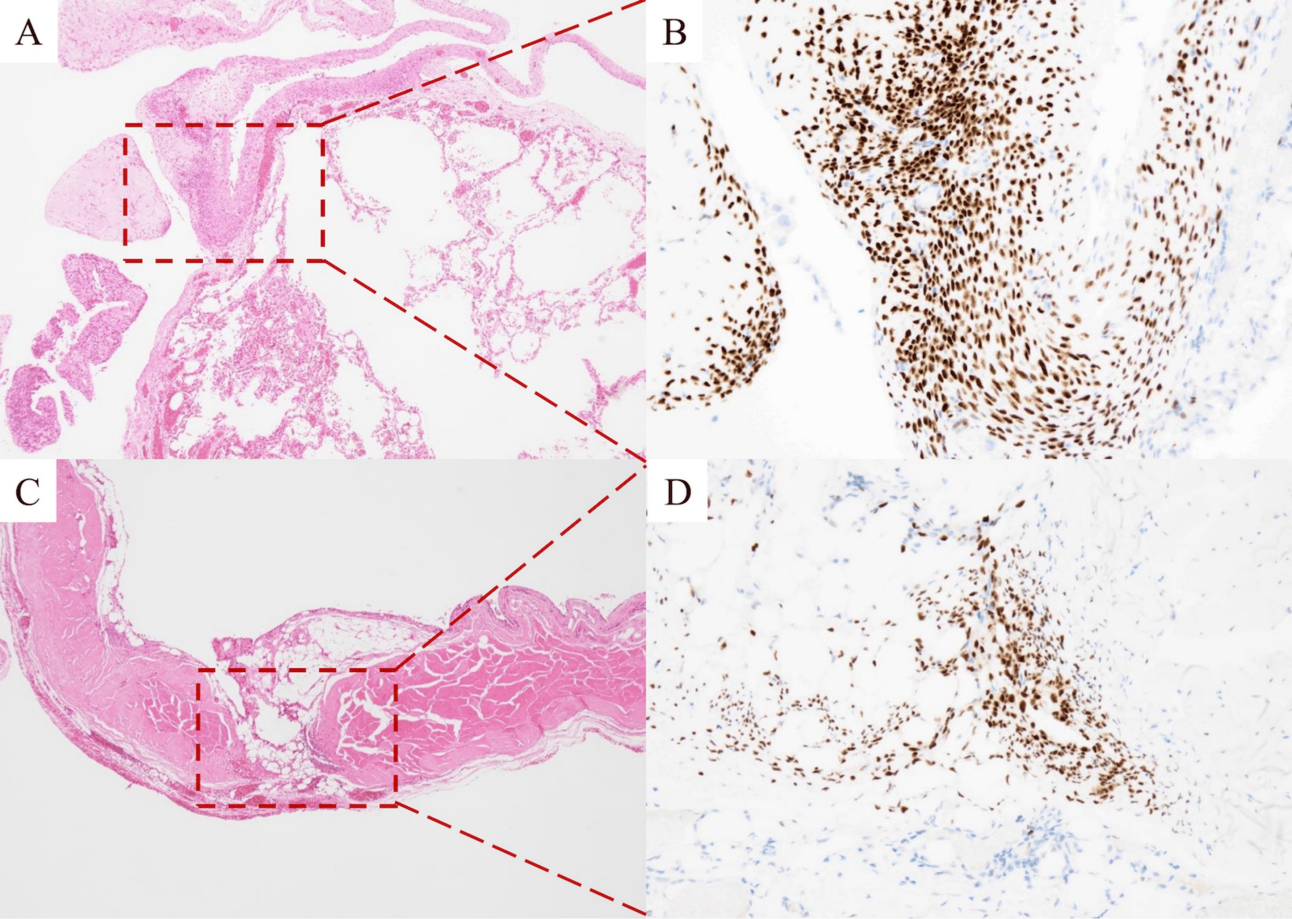
Histopathological findings (A and B: bulla; C and D: diaphragm). A: Low-power microscopic view of the bulla (hematoxylin and eosin staining). B: Cells in the bulla wall positive for anti-estrogen receptor antibody. C: Low-power microscopic view of the diaphragm (hematoxylin and eosin staining). D: Cells in the diaphragm positive for anti-estrogen receptor antibody.

Pathological examination revealed small clusters of spindle-shaped cells in the lung cyst and diaphragm. Immunohistological staining was positive for estrogen and progesterone receptors, confirming a diagnosis of catamenial pneumothorax (Figure 3).

After discharge, she was prescribed dienogest 1 mg/day by the gynecology department and has remained recurrence-free for over three years.

## Discussion

Endometriosis is categorized by location into common, less common, and rare sites, with the latter two comprising roughly 2% of cases. Endometriosis involving the lungs and pleura is classified as thoracic endometriosis [1]. Pneumothorax, which occurs during the menstrual cycle is called catamenial pneumothorax and occurs mainly in the reproductive age. As this is an estrogen-mediated disease, it rarely occurs in menopausal women; however, tamoxifen acts as an agonist on the endometrium and may therefore be a risk factor for the development of this disease even after menopause [4].

Two theories have been reported as the main pathogenesis of thoracic endometriosis: the intraperitoneal origin theory [2] and the hematogenous metastasis theory [3]. In the intraperitoneal origin theory, the flow of ascites is thought to be clockwise, and endometrial cells flowing with the flow accumulate on the diaphragmatic surface of the liver and attach to the tendon center, causing repeated pneumothorax during menstruation. In the hematogenous metastasis theory, endometrial cells enter the bloodstream during surgical procedures such as abortion and migrate via the transvenous route through the pulmonary artery to the lungs, causing recurrent pneumothorax during menstruation. When endometrial cells grow on the cyst wall, pneumothorax develops with a rupture of the cyst wall during menstruation [5].

Alternatively, if endometrial tissue engrafts within normal lung parenchyma, it may present as an intrapulmonary shadow or nodule with symptoms of hemoptysis or bloody sputum [6,7]. In the theory of intraperitoneal origin, the affected side is mainly the right side. For intraperitoneal origin cases, the affected side is typically the right. However, hematogenous metastasis theoretically involves both sides equally due to the pulmonary artery distribution. However, since catamenial pneumothorax are right-sided and have diaphragmatic lesions, the intraperitoneal origin theory is the majority. The diaphragmatic lesion is more likely to be of intraperitoneal origin, but since there is also pulmonary involvement, it cannot be ruled out, although hematogenous metastasis is most likely in both cases when considered as a single entity. Preoperative CT showed no findings other than pulmonary cysts, and at the time of surgery, the patient was considered to have a spontaneous pneumothorax. The CT images of the cyst wall were reviewed postoperatively, and no wall thickening was observed, making preoperative identification of endometrial tissue difficult.

In cases of catamenial pneumothorax without pulmonary cysts, surgery is diagnostic and involves partial resection of the lung and diaphragm with erythema, small holes, and hemorrhagic spots. The small hole is formed in the vulnerable center of the tendon, and the enlargement of the hole may cause intraperitoneal organs to become trapped [8]. Preoperative CT should be performed to confirm the presence of a bulging lesion on the diaphragm and reconstruction of the diaphragm should be considered [9,10]. If the intraoperative findings show no diaphragmatic lesions, only pulmonary cysts, and no erythema or hemorrhagic spots, it is likely to be misinterpreted as a spontaneous pneumothorax. In this case, there was a small hole in the diaphragm, but no erythema suspicious of hemorrhage and no cyst wall. In such cases, immunostaining for estrogen and progesterone receptors on resected specimens can confirm the diagnosis. Histologic findings of endometrial tissue have been reported in 23.1% of premenopausal women undergoing pneumothorax surgery [11] and immunohistology testing for female hormones should be actively considered.

One of the treatments for pneumothorax is pleurodesis; however, hormone therapy is typically prioritized in the management of catamenial pneumothorax. Pleurodesis is generally considered for refractory cases where pneumothorax recurs repeatedly despite hormonal therapy. In the present case, pleurodesis was not performed, as there was no recurrence following hormonal treatment.

The main treatment for this disease is hormone therapy, which has been reported to have a lower recurrence rate than surgery alone [12-14]. Hormone therapy by a gynecologist is essential because the disease is difficult to cure with surgery alone. Hormonal therapy often includes gonadotropin-releasing hormone (GnRH) agonists, danazol, and LEP preparations, as in the case of endometriosis. In the present case, the patient developed dysmenorrhea while taking an LEP preparation for the treatment of dysmenorrhea. This drug acts on the pituitary gland to suppress follicle development and ovulation. It is also intended to relieve menstrual symptoms by stabilizing the menstrual cycle and inhibiting prostaglandin production [15]. Normally, the drug is taken for 21 days and then withdrawn for seven days, so there is a small amount of blood loss during the withdrawal period and catamenial pneumothorax may occur. However, pneumothorax may develop during ovulation and cannot be diagnosed based on the menstrual cycle alone [16]. Usually, catamenial pneumothorax is reported to occur more frequently within 24 hours before the onset of menstruation than within 72 hours after the onset of menstruation [17]. In the present case, the onset was on the third day of LEP drug withdrawal, so we suspected catamenial pneumothorax and operated on the patient. Although endometrial tissue was present in the cyst wall and diaphragm, there was no erythema or bleeding spots and only a small hole in the diaphragm, which was thought to be due to the low endogenous hormone level suppressed by the LEP drug and the low endometrial tissue itself. Even if menstrual symptoms and menstrual blood volume are reduced after taking LEP, this disease cannot be ruled out and must be included in the differential diagnosis of a right pneumothorax in a woman.

## Conclusions

This case highlights the rare occurrence of catamenial pneumothorax in a patient with a long history of endometriosis and cyclic LEP use. The presence of estrogen- and progesterone-receptor-positive cells in the lung and diaphragm strongly suggests a hormonal influence on pneumothorax recurrence. Clinicians should be aware of the potential for catamenial pneumothorax in patients with endometriosis who present with unexplained chest symptoms, especially around menstrual cycles or hormonal therapy changes. Early recognition, appropriate surgical intervention, and postoperative hormonal management can prevent recurrence and improve patient outcomes.
